# DiDyNet: a robust framework for differential dynamic network inference from longitudinal multi-omics data

**DOI:** 10.1093/bioadv/vbag192

**Published:** 2026-07-30

**Authors:** Zhe Liu, Kesong Wu, Taesung Park

**Affiliations:** Interdisciplinary Program in Bioinformatics, Seoul National University, Seoul, 08826, Republic of Korea; Ian Frazer Centre for Children’s Immunotherapy Research, The University of Queensland, St Lucia, Queensland 4072, Australia; Interdisciplinary Program in Bioinformatics, Seoul National University, Seoul, 08826, Republic of Korea; Department of Statistics, Seoul National University, Seoul, 08826, Republic of Korea

## Abstract

**Motivation:**

Understanding disease dynamics from longitudinal multi-omics is hindered by traditional approaches that focus on univariate trajectories and static networks while ignoring temporal evolution. We developed DiDyNet, a framework for identifying phenotype-specific temporal molecular networks by defining dynamic coupling as coordinated molecular trajectories. DiDyNet operates through four steps: (i) two-dimensional variance-based filtering to prioritize dynamic features; (ii) quantification of subject-specific coordination using Dynamic Time Warping to accommodate asynchrony; (iii) statistical testing for differential dynamic couplings; and (iv) linear mixed model-based post-hoc refinement to distinguish genuine coordinated dynamics from stochastic noise.

**Results:**

Simulation studies showed that DiDyNet significantly outperformed static summary statistics, including the mean, median, and difference, which cannot capture dynamic signals. Dynamic Time Warping-based quantification also demonstrated greater robustness than Euclidean distance, correlation-based distance, and constrained alignment methods under temporal misalignment and signal sparsity. Application to an insulin resistance cohort identified a coordinated cross-omics network linking systemic inflammation with intracellular stress responses.

**Availability:**

Source code is freely available at https://github.com/bioinfoliu/DiDyNet.

## Introduction

With the rapid advancement of high-throughput omics technologies, researchers can now simultaneously measure molecular states across multiple layers, including the transcriptome, proteome, metabolome, and cytokine profiles (Hasin *et al*. 2017). These multi-omics datasets reveal complex interactions among different regulatory levels within biological systems and have become a cornerstone of systems biology research. In particular, the increasing adoption of longitudinal clinical studies has generated datasets in which repeated measurements are collected from the same subjects over multiple time points. Compared with static snapshots, longitudinal multi-omics studies offer unprecedented opportunities to capture time-dependent molecular changes underlying disease progression, stratification, and treatment response. Understanding these dynamic mechanisms, especially in complex diseases such as type 2 diabetes (T2D) and cancer, remains a central challenge in modern biomedical research. However, uncovering system-level mechanisms from such data remains challenging, as there is a lack of interpretable computational frameworks designed to capture the dynamic rewiring of molecular networks.

Traditional differential expression (DE) analyses aim to identify individual molecular features that significantly differ between conditions. While widely adopted, these approaches treat each feature independently, overlooking the complex interactions and dependencies among molecules that govern biological function (Smyth 2004, Robinson *et al*. 2010, Love *et al*. 2014).

To address this limitation, differential network analysis has emerged as a powerful complementary strategy, particularly for static omics data. Such methods infer and compare molecular interaction networks across conditions, revealing how regulatory or physical interactions are rewired in disease, development, or environmental responses (De La Fuente 2010, Ideker and Krogan 2012). Despite their utility in capturing systems-level alterations, most differential network approaches were designed for cross-sectional data. Prominent frameworks, such as WGCNA (Langfelder and Horvath 2008) and DGCA (McKenzie *et al*. 2016), have been widely adopted to identify stable co-expression modules and differentially correlated pairs between phenotypic groups. However, despite their robustness in analysing cross-sectional data, these approaches inherently treat samples as independent snapshots. When applied to longitudinal studies, these approaches typically compare networks derived from summary statistics, such as averages or endpoints, between groups but fail to capture the temporal dynamics of molecular interactions. Despite these advances, a critical methodological gap remains. Longitudinal multi-omics data, in which molecular profiles are repeatedly measured over time within individuals, introduce new analytical challenges: how do molecular trajectories evolve over time, and do these trajectories differ across phenotypic groups? While repeated measures ANOVA and linear mixed models (LMMs) (Park *et al*. 2003, Vestal *et al*. 2022) have improved the analysis of longitudinal omics data, they are primarily designed for single-omics studies and model each feature independently. Consequently, they are limited in capturing coordinated temporal patterns or shared dynamics within or across omics layers, thereby restricting the identification of complex molecular processes that underlie disease progression.

To address this gap, we developed DiDyNet, a novel framework for identifying differential dynamic networks from longitudinal multi-omics data. Unlike conventional DE approaches that focus on marginal feature changes, DiDyNet integrates Dynamic Time Warping (DTW) with a rigorous post-hoc refinement strategy to capture asynchronous and time-evolving molecular patterns. Inspired by differential network analysis, it identifies rewired molecular pairs whose dynamic coordination differs significantly between groups, revealing regulatory dysfunction often missed by static methods (Zeng *et al*. 2013).

We validated DiDyNet through extensive simulation studies, demonstrating its superiority over conventional metrics (e.g. Euclidean distance and Pearson correlation) in identifying genuine differential dynamic couplings (DDCs), particularly under conditions of temporal misalignment and noise. Application to a longitudinal multi-omics cohort comparing insulin-resistant and insulin-sensitive individuals revealed intricate cross-omics networks enriched in pathways implicated in insulin resistance (IR). In summary, we present DiDyNet as a scalable and statistically rigorous framework for uncovering coordinated molecular dynamics, providing a powerful tool for systems-level understanding of disease progression and regulation.

## Methods

**Figure 1:**
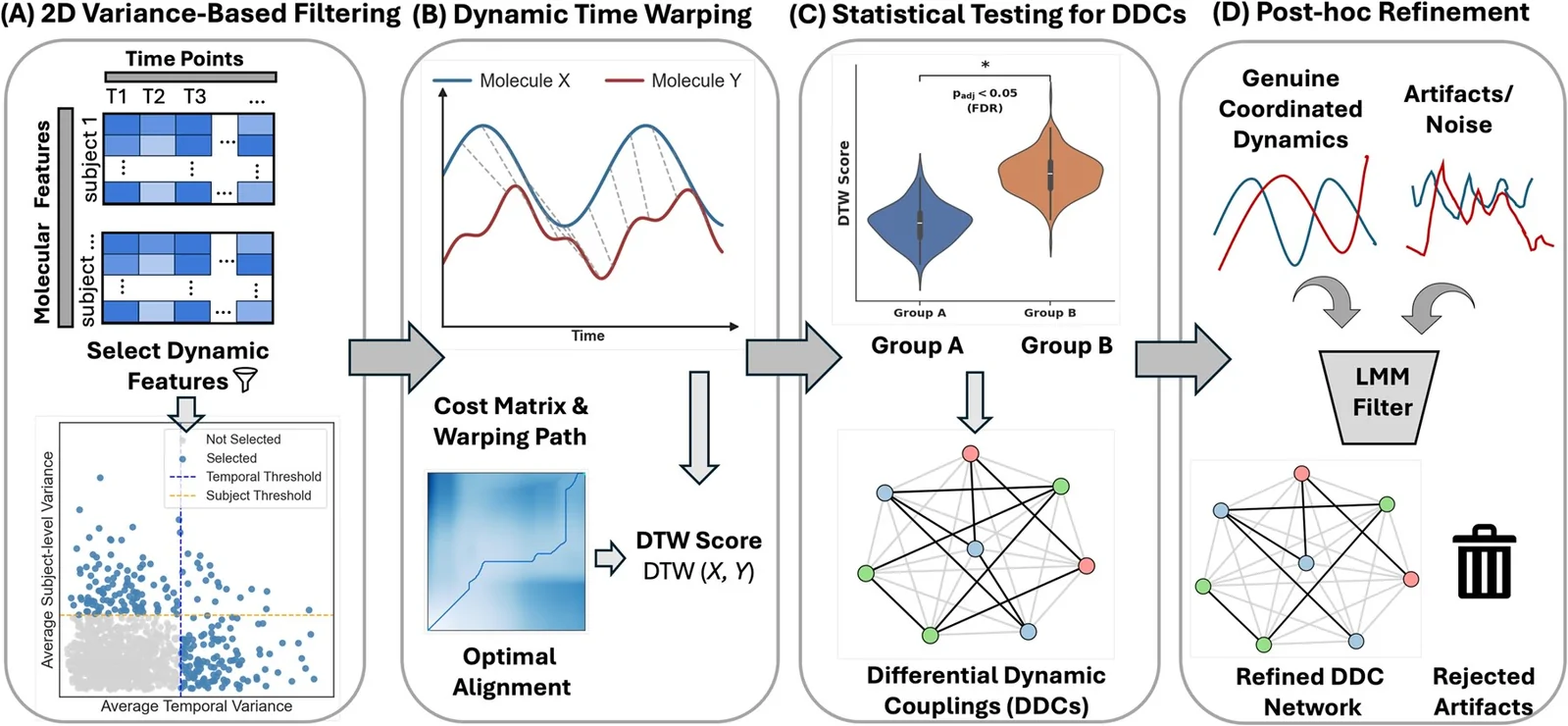
The DiDyNet Framework for Differential Dynamic Network Inference. (A) Preprocessing and two-dimensional variance filtering: raw time-series features are preprocessed, and low-quality or noisy features are removed. (B) *Subject-specific trajectories*: feature profiles are compiled for each subject. (C) *DTW alignment and initial testing*: feature trajectories are aligned using DTW, followed by Wilcoxon rank-sum tests with Benjamini–Hochberg false discovery rate correction to identify DDCs. (D) *Post-hoc refinement*: significant DDCs are categorized into three types according to whether individual feature-level effects are significant for one, both, or neither of the paired features. (E) *Differential dynamic network*: the inferred network visualizes relationships between molecular features and highlights key hubs.

DiDyNet establishes a unified analytical framework that integrates three key methodological innovations, as outlined in the workflow shown in Fig. 1. First, a two-dimensional (2D) variance-based filtering strategy improves computational efficiency and enhances the signal-to-noise ratio by prioritizing molecular features that display both temporal variability and between-group divergence. Second, it quantifies DC among molecular trajectories using DTW, enabling the identification of non-linear and asynchronous temporal dependencies that are inherent to longitudinal omics data. Finally, a post-hoc refinement procedure discriminates genuine coordinated molecular dynamics from artifacts arising from individual feature fluctuations, ensuring that the inferred associations represent true biological coordination.

### Feature filtering based on variance components

To reduce the dimensionality of the longitudinal omics data and select features with significant dynamic variation, we implemented a 2D variance-based filtering strategy. We consider a longitudinal dataset with *n* subjects and *t* time points, where $y_{ij}$ denotes the observation from subject *i* (i=1,…,n) at time point *j* (j=1,…,t). Our filtering approach considers two sources of variation. The first is the Average Temporal Variance, which measures the average dispersion of observations over time within each subject. This captures the intra-subject dynamics and is calculated as


(1) $$V_{t}=\frac{1}{n}\sum_{i=1}^{n}\left(\frac{1}{t-1}\sum_{j=1}^{t}{\left(y_{ij}-{\bar{y}}_{i\cdot }\right)}^{2}\right),$$

where ${\bar{y}}_{i\cdot }$ denotes the subject-wise mean across all time points. The second is the Average Subject-level Variance, which measures the average dispersion of observations across subjects at each time point. This captures inter-subject heterogeneity and is calculated as


(2) $$V_{s}=\frac{1}{t}\sum_{j=1}^{t}\left(\frac{1}{n-1}\sum_{i=1}^{n}{\left(y_{ij}-{\bar{y}}_{\cdot j}\right)}^{2}\right),$$

where ${\bar{y}}_{\cdot j}$ denotes the time-point mean across all subjects. Features were selected for downstream analysis if they exhibited high values in either or both variance components, ensuring that we retained features with substantial dynamic changes over time or significant variability across the study population. While this filtering step was applied in the analysis of real datasets, it was omitted in the simulation studies because the number of features was small and such preprocessing was unnecessary.

### Dynamic coupling quantification and statistical testing

In this study, *dynamic coupling* (DC) refers to the extent to which the temporal trajectory of one molecule ($X_{h}$) follows or matches that of another molecule ($X_{g}$), thereby capturing the observable coordination between their temporal patterns. This definition focuses on trajectory similarity rather than underlying mechanistic interactions. Consequently, the observed “following” relationship may arise from direct regulation, shared upstream regulators, or more complex feedback mechanisms, although our framework does not attempt to distinguish among these possibilities. It should also be noted that the definition of DC adopted here differs from that used in some previous studies, where the term has been interpreted as mechanistic coupling or network connectivity.

To quantify temporal coordination, we employ DTW, which is particularly well suited for longitudinal omics data because it accommodates temporal asynchrony while capturing nonlinear trajectory patterns that are often missed by methods based on linear correlations. Biological responses frequently exhibit delayed effects between changes in $X_{h}$ and the corresponding responses in $X_{g}$, and DTW naturally accounts for such temporal shifts by identifying the optimal alignment between trajectories. Moreover, DTW is robust to irregular or sparse sampling because it does not require observations to occur at identical time points or even for time series to have equal lengths. Consequently, a smaller DTW distance indicates stronger DC, reflecting greater similarity between the temporal trajectories of $X_{h}$ and $X_{g}$.

To identify feature pairs exhibiting coordinated dynamic changes across different omics layers, we first compute the pairwise DTW distance between features *g* and *h* for each subject *i*:


(3) $$\mathrm{DTW}\left(f_{i,g}^{s},f_{i,h}^{s}\right)=\underset{\omega \in W}{\min}\sum_{(t_{1},t_{2})\in \omega }{\left(f_{i,g}^{s}(t_{1})-f_{i,h}^{s}(t_{2})\right)}^{2},$$

where $f_{i,g}^{s}(t)$ denotes the longitudinal trajectory of feature *g* for subject *i* in group *s*, *W* represents the set of all valid warping paths, and ω denotes a specific alignment path matching time points $t_{1}$ and $t_{2}$.

Identification of DDCs consists of three steps. First, pairwise DTW distances are calculated between every feature pair for each subject within each phenotypic group. Second, these subject-level DTW distances are aggregated to generate two group-specific distributions for each feature pair. Finally, a nonparametric Wilcoxon rank-sum test is performed to evaluate the null hypothesis.


(4) $$H_{0}:\mathrm{median}\left[\mathrm{DTW}\left(f_{g}^{A},f_{h}^{A}\right)\right]=\mathrm{median}\left[\mathrm{DTW}\left(f_{g}^{B},f_{h}^{B}\right)\right].$$

Rejecting $H_{0}$ indicates that the DC between features *g* and *h* differs significantly between the two phenotypic groups. A smaller median DTW distance in one group indicates stronger DC, reflecting greater similarity between temporal trajectories than that observed in the other group. To account for the large number of pairwise comparisons, *P*-value were adjusted using the Benjamini–Hochberg false discovery rate (FDR) procedure, and adjusted *P *< .05 was considered statistically significant.

### Post-hoc refinement using linear mixed models

The post-hoc refinement procedure is essential for analysing real-world longitudinal multi-omics data, where substantial technical noise, sparsity, and stochastic fluctuations may confound the interpretation of DC. Although DTW explicitly models potentially nonlinear temporal relationships between feature trajectories and is therefore used to quantify DDCs, its flexible warping mechanism may occasionally align noise-driven fluctuations, resulting in spurious coupling signals. To address this limitation, we implemented a post-hoc filtering and categorization framework in which LMMs are not used to quantify nonlinear coupling itself, but rather to characterize the individual temporal behavior of each feature as a conservative baseline filter.

For each significant DDC pair (g,h), we independently modeled the longitudinal trajectories of features *g* and *h* using an LMM with fixed effects for group, time, and their interaction, together with a subject-specific random intercept. Time was treated as a continuous variable to evaluate overall differences in temporal trends. Specifically, for each feature *X*, the following model was fitted:


(5) $$X_{ij}=\beta_{0}+\beta_{1}{\text{Group}}_{i}+\beta_{2}{\text{Time}}_{ij}+\beta_{3}\left({\text{Group}}_{i}\times {\text{Time}}_{ij}\right)+b_{0i}+\varepsilon_{ij},$$

where $b_{0i}$ denotes the subject-specific random intercept and $\varepsilon_{ij}$ represents the residual error. In this model, $\beta_{1}$ captures the overall group effect, $\beta_{2}$ represents the overall linear temporal trend, and $\beta_{3}$ quantifies the differential temporal trend between phenotypic groups. Statistical significance of the interaction term was assessed using a likelihood ratio test. We emphasize that this linear modeling step serves solely as a conservative baseline filter rather than an approximation of nonlinear coupling. DTW and LMM therefore play complementary roles within the proposed framework: DTW captures potentially nonlinear inter-feature temporal relationships, whereas LMM filters out apparent coupling signals that can be explained by simple linear group differences at the individual-feature level.

Based on the combined results of the DDC test and the individual LMM analyses, significant DDC pairs were classified into three interpretative categories:

1. *Subtle Coordinated Coupling (SCC):* Significant DDCs for which neither feature exhibits a significant individual group difference, suggesting genuine rewiring of coordinated temporal dynamics.
2. *Unilateral Driver DDC:* Significant DDCs for which only one feature exhibits a significant individual group difference, indicating that the observed coupling change is primarily driven by a strong marginal effect.
3. *Both-Driven DDC:* Significant DDCs for which both features exhibit significant individual group differences, suggesting that the observed coupling likely reflects parallel responses to a shared upstream regulator or biological process.

Only SCC pairs were retained for downstream network construction and biological interpretation, ensuring that the resulting differential dynamic network emphasizes subtle systems-level regulatory rewiring that would otherwise be obscured by dominant individual feature changes.

### Construction and analysis of the differential dynamic network

The filtered set of high-confidence SCC pairs was used to construct an undirected differential dynamic network, in which nodes represent molecular features and edges represent significant SCC relationships. To identify key regulatory elements within the network, hub features were defined based on their connectivity using degree centrality, that is, the number of edges connected to each node. Features ranked among the highest by degree centrality were designated as network hubs for downstream biological interpretation.

### Implementation and computational efficiency

All computational analyses were performed on a Linux server equipped with dual Intel Xeon Silver 4216 processors (32 physical cores, 64 logical cores, 2.10 GHz) and approximately 700 GB of memory. A single end-to-end analysis of the real longitudinal multi-omics dataset required approximately 7 hours, with the majority of the computational cost arising from DTW calculations.

For the subsequent 100-run robustness analysis, pairwise DTW distances for every subject-feature pair were cached after their initial computation, thereby avoiding redundant calculations across repeated analyses. This optimization reduced the overall computational burden, allowing the complete robustness analysis to be completed in approximately 13 hours.

## Results

To systematically evaluate the effectiveness of our DiDyNet framework for differential dynamic network analysis, we performed both simulation studies and real-world data applications. The simulation study aimed to assess the method’s ability to accurately identify changes in molecular coupling patterns, thereby evaluating the discriminative performance, statistical power, and robustness of the DTW-based detection under varying conditions. The real data analysis, conducted using a longitudinal multi-omics cohort, was designed to demonstrate the biological relevance of the identified DDCs. In particular, we examined whether the hub genes within the constructed differential dynamic networks were enriched for pathways and processes consistent with previously reported biological mechanisms.

### Simulation study

#### Simulation setup

To systematically evaluate the performance of DiDyNet in detecting DDCs, we conducted a series of simulation experiments. Synthetic longitudinal datasets were generated under a generative model specifically designed to produce feature pairs exhibiting group-specific coupling structures. Under the default simulation setting, we generated p=30 features for n=20 subjects per group across T=8 time points. Two phenotypic groups (e.g. Case and Control) were simulated, with five feature pairs designated as true DDCs. Gaussian noise with variance $\sigma^{2}=0.1$ was added to the simulated trajectories. The remaining features were generated independently from first-order autoregressive [AR(1)] processes with autoregressive coefficient ϕ=0.8 and served as null pairs.

For each simulated DDC pair (g,h), group-specific coupling was generated according to the following linear mixing model:


(6) $$f_{i,h}^{s}(t)=\rho_{s}\left[T\left(f_{i,g}^{s}(t)\right)+\varepsilon_{i}(t)\right]+(1-\rho_{s})\eta_{i}(t),$$

where $\rho_{s}$ denotes the group-specific coupling strength (e.g. $\rho_{A}=0.9$ and $\rho_{B}=0.1$). The transformation function T(·) was randomly selected from a predefined collection of nonlinear functions (Table S1). The additive noise term $\varepsilon_{i}(t)$ was sampled from a Gaussian distribution,


$$\varepsilon_{i}(t)∼N(0,\sigma^{2}),$$

whereas $\eta_{i}(t)$ represents background noise generated from an AR(1) process. To mimic the asynchronous nature of biological processes, a random temporal lag *l* was introduced into feature *h*, where


$$l∼\text{Uniform}(0,L_{\max}),$$

with the default value $L_{\max}=3$. Furthermore, to emulate the sparse and irregular sampling commonly observed in longitudinal clinical studies, 10% of time points were randomly removed from each simulated trajectory during data generation. Consequently, the resulting input data consisted of unequal-length time series. Prior to DTW distance calculation, all trajectories were standardized using *Z*-score normalization.

To further evaluate the robustness of DiDyNet, we performed a comprehensive sensitivity analysis by varying one simulation parameter at a time while holding all remaining parameters at their default values. The range of parameter settings evaluated is summarized in Table S2.

#### Baseline methods

To rigorously evaluate the effectiveness of the proposed DTW-based framework for detecting DDCs under temporal distortions, we compared DiDyNet with five alternative distance metrics. Specifically, (i) Euclidean distance was used as the conventional point-to-point baseline; (ii) Pearson and Spearman correlation coefficients were included to measure linear and monotonic associations, respectively, with correlation coefficients (*r*) converted into dissimilarity scores using 1−|r|; (iii) Time-Lagged Cross-Correlation (TLCC) was employed to alleviate the sensitivity of Pearson correlation to temporal phase shifts, thereby providing a baseline for lagged interactions; and (iv) Constrained Dynamic Time Warping (cDTW), which restricts the warping path using a Sakoe–Chiba window of size 2, was included to evaluate the effect of limiting temporal alignment flexibility under irregular sampling. Because Euclidean distance, Pearson correlation, Spearman correlation, and TLCC require equal-length input sequences, linear interpolation was applied to irregularly sampled trajectories before distance calculation.

To quantify the benefit of modeling complete temporal trajectories rather than reducing longitudinal data to static summaries, we further compared DiDyNet with three static feature representations: (i) the Mean Intensity approach, which calculates the difference between the average values of two time series, thereby ignoring temporal dynamics; (ii) the Endpoint Difference method, which compares the change between the initial and final time points to capture only the overall trend; and (iii) the Midpoint strategy, which assesses values solely at the median time point. These static baselines were included to assess whether modeling the complete temporal evolution of molecular trajectories provides greater discriminative power than conventional summary statistics.

#### Evaluation metrics

We evaluated DiDyNet’s discriminative performance using the area under the receiver operating characteristic curve (AUC), aggregated across 1000 independent simulation runs. Crucially, to filter stochastic variance inherent in complex longitudinal data, DiDyNet scores candidate interactions using non-parametric statistical confidence rather than raw distance metrics. Specifically, after computing DTW distances, the final score for each pair is defined as the *P*-value derived from a Wilcoxon rank-sum test comparing the strongly (Group A) and weakly (Group B) coupled states. These confidence scores were then used to calculate the AUC:


(7) $$\text{AUC}=\int_{0}^{1}\text{TPR}(x)d\left(\text{FPR}(x)\right),$$

where TPR and FPR denote the true positive rate and false positive rate, respectively. The final reported AUC corresponds to the average AUC over 1000 independent simulation runs, where values closer to 1 indicate superior performance in distinguishing true DDCs from null feature pairs.

#### Performance under varying simulation conditions

**Figure 2:**
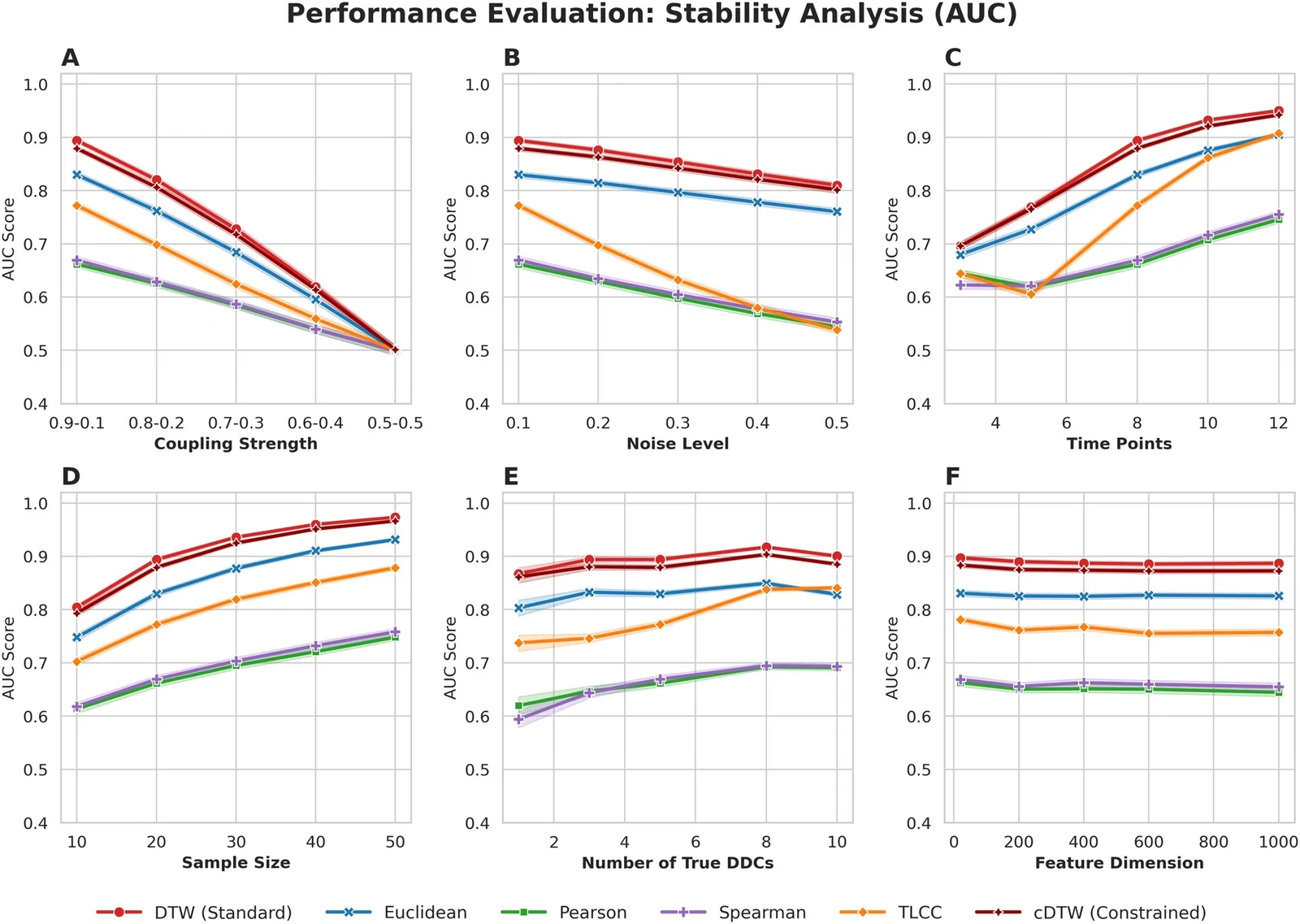
Performance comparison of coupling detection methods under varying simulation conditions. Performance was evaluated using the pooled area under the receiver operating characteristic curve (AUC) across 1000 independent simulation runs. Sensitivity analyses were performed by varying (A) coupling strength, (B) noise level, (C) number of time points, (D) sample size, (E) number of true DDCs, and (F) feature dimensionality. DiDyNet (DTW; red) was compared with Constrained Dynamic Time Warping (cDTW; dark red), Time-Lagged Cross-Correlation (TLCC; orange), interpolated Pearson correlation (green), interpolated Spearman correlation (purple), and interpolated Euclidean distance (blue).

We systematically compared the performance of DiDyNet with four baseline methods (Euclidean distance, Pearson correlation, Spearman correlation, and Time-Lagged Cross-Correlation) as well as cDTW. Across nearly all simulation scenarios, DiDyNet consistently achieved the highest discriminative accuracy. As illustrated in Fig. 2, the DTW-based methods consistently outperformed competing approaches, particularly under challenging conditions where conventional distance metrics failed to capture dynamic temporal associations.

To evaluate robustness to signal quality, we systematically varied both the contrast in coupling strength between the two phenotypic groups and the level of observational noise. As expected, the performance of all methods gradually declined as the difference in coupling strength between Group A and Group B decreased (Fig. 2A). Nevertheless, DiDyNet consistently achieved the highest AUC across all contrast levels until the signal became indistinguishable. Similarly, increasing noise levels (Fig. 2B) resulted in performance degradation for all methods, although DiDyNet exhibited the slowest decline. These findings suggest that the trajectory-based alignment mechanism of DTW is inherently more robust to random amplitude fluctuations than conventional point-wise similarity measures.

We further investigated the influence of sequence length, cohort size, signal density, and feature dimensionality on detection performance. As the number of time points increased from 3 to 12 (Fig. 2C), the AUC of all methods improved, with DiDyNet exhibiting the greatest performance gain. Similarly, DiDyNet consistently achieved the highest AUC across all cohort sizes, including small cohorts containing only 10 subjects per group, while maintaining stable performance as sample size increased (Fig. 2D). To assess robustness to varying signal densities, we increased the number of true SCC pairs while holding all remaining simulation parameters constant (Fig. 2E). DiDyNet maintained consistently high performance across all conditions, demonstrating its ability to detect varying numbers of true dynamic couplings without degradation. Finally, we evaluated scalability under high-dimensional settings by increasing the total number of simulated features to 1000 (Fig. 2F), thereby approximating the dimensionality of typical high-throughput omics datasets. To maintain computational tractability while preserving statistical validity, all true DDC pairs were evaluated together with a randomly sampled set of null feature pairs proportional to the feature dimension (10×p). Under these conditions, DiDyNet exhibited virtually no loss in performance and consistently achieved the highest AUC among all competing methods.

Collectively, these results demonstrate that DiDyNet is robust to temporal noise, signal sparsity, limited sample size, and high-dimensional feature spaces, highlighting its scalability and effectiveness for longitudinal multi-omics studies.

#### Comparison with static feature representations

**Figure 3:**
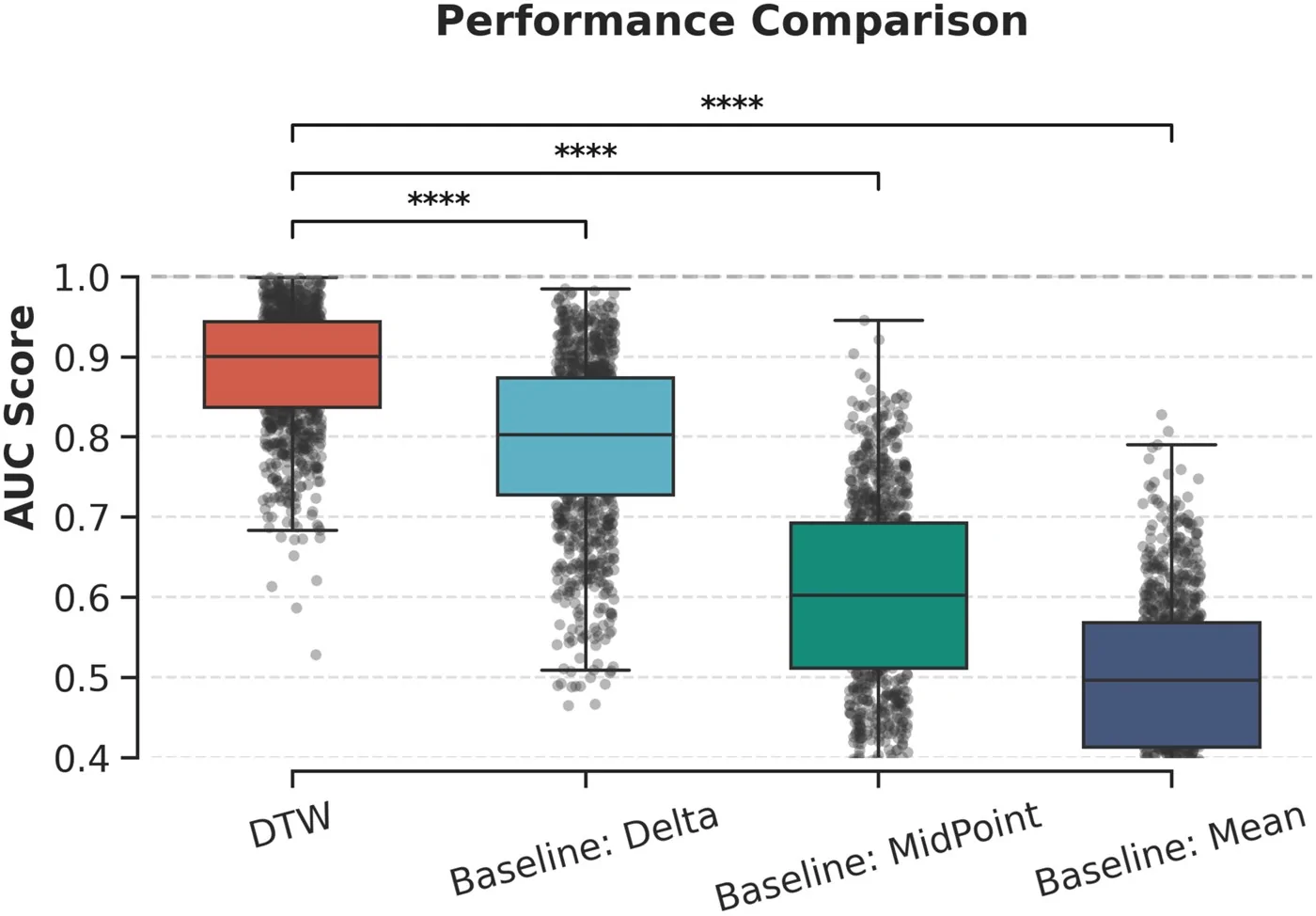
Comparison of classification performance between DiDyNet and static feature representations. Distribution of AUC values obtained from 1000 independent simulation runs for DiDyNet (DTW) and three static feature representations. DiDyNet consistently achieved the highest discriminative performance across all simulations. Brackets indicate statistical significance based on the Wilcoxon rank-sum test.

To evaluate the importance of modeling complete temporal trajectories rather than relying on static summary statistics, we compared DiDyNet with three conventional static feature representations. As shown in Fig. 3, the proposed DTW-based framework consistently outperformed all static baselines across 1000 independent simulation runs. DiDyNet achieved the highest discriminative performance, with a mean AUC of 0.885 and the smallest variability (standard deviation = 0.074), indicating both high accuracy and stable performance.

Among the static baselines, the Endpoint Difference method, which measures the difference between the first and last observations, achieved moderate performance (mean AUC = 0.792). This result suggests that the overall temporal trend contains partial discriminative information but fails to capture the intermediate temporal dynamics recovered by DTW. In contrast, the Midpoint Value (mean AUC = 0.595) and Mean Intensity (mean AUC = 0.493) representations exhibited substantially lower performance, with the Mean Intensity approach performing close to the random-classification level.

These findings demonstrate that discriminative DC is primarily encoded in the complete temporal evolution of molecular trajectories. Consequently, reducing longitudinal time series to static summary statistics results in a substantial loss of information, highlighting the importance of explicitly modeling temporal dynamics when identifying DDCs.

### Real data application

#### Data description and preprocessing

We applied DiDyNet to a publicly available longitudinal multi-omics dataset originally reported by Sailani *et al*. (2020), which contains one year of follow-up measurements across multiple molecular layers. The dataset comprises cytokine, proteomic, and transcriptomic profiles collected from human subjects at irregularly spaced time points.

To ensure consistency across downstream analyses, only subjects with clearly defined insulin-resistant (IR) or insulin-sensitive (IS) phenotypes were retained. Duplicate measurements obtained at the same visit were averaged for each subject. The complete preprocessing workflow, including phenotype filtering, duplicate averaging, and the progressive selection of subjects shared across all omics layers, is summarized in Fig. S1.

**Figure 4:**
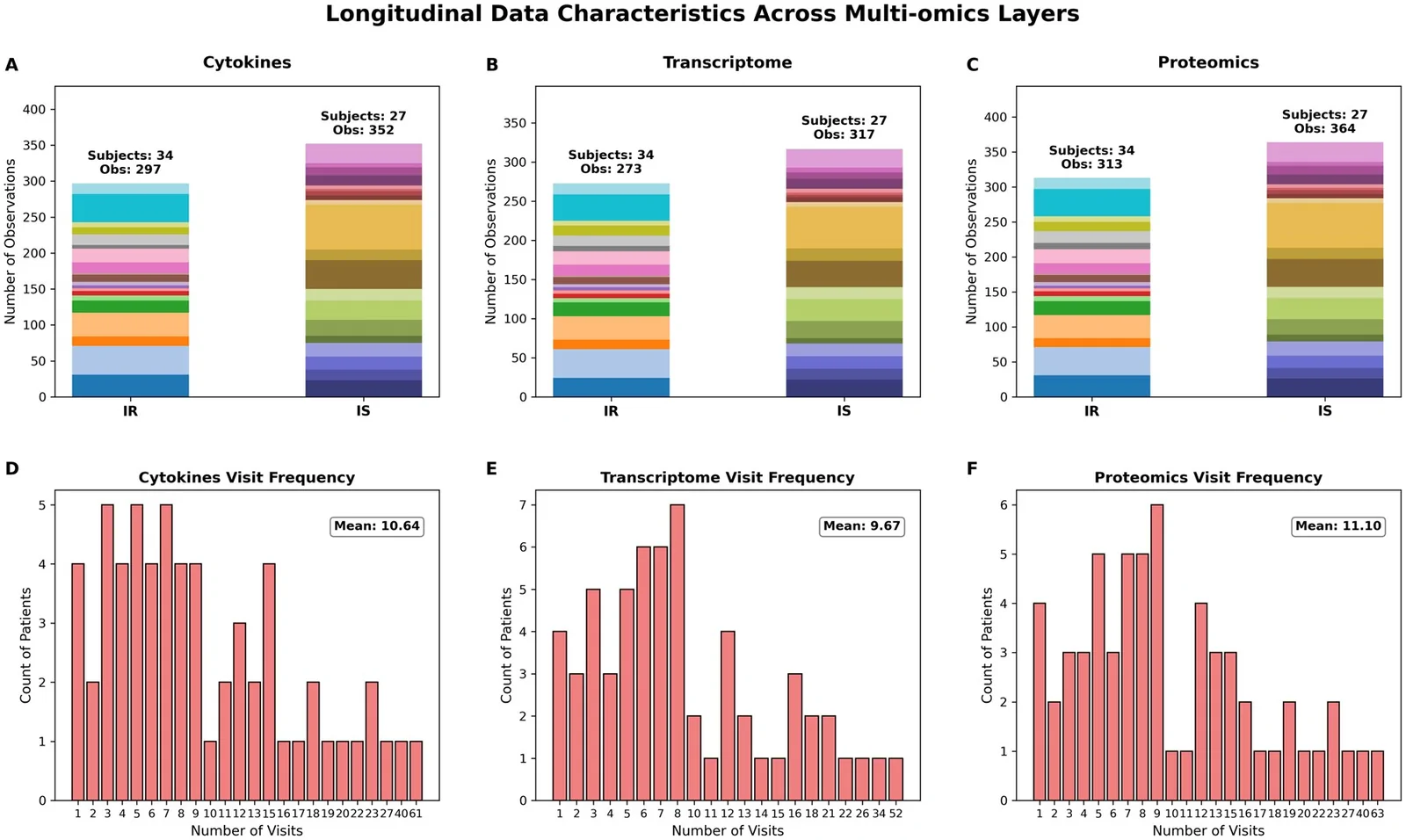
Distribution of longitudinal observations and visit frequencies across multi-omics layers. (A–C) Stacked bar charts showing the total numbers of longitudinal observations in the insulin-resistant (IR) and insulin-sensitive (IS) groups for the cytokine (A), transcriptomic (B), and proteomic (C) datasets. Each bar is subdivided according to individual subjects. (D–F) Histograms showing the distributions of longitudinal visit frequencies per subject for the corresponding omics layers. Bars indicate the numbers of subjects with each visit frequency, and the average numbers of visits per subject are highlighted in the callout boxes.

After preprocessing, 61 subjects remained with complete multi-omics data. Within this cohort, 649, 677, and 590 longitudinal observations were retained for the cytokine, proteomic, and transcriptomic datasets, respectively. The availability of multi-omics measurements for each subject across all visits is illustrated in Fig. S2. Longitudinal sampling patterns and visit frequencies are summarized in Fig. 4, showing an average of approximately 10 visits per subject, thereby providing sufficient temporal resolution for dynamic network inference. Finally, all molecular features were standardized using Z-score normalization implemented with the StandardScaler algorithm to ensure comparability across heterogeneous omics layers (Pedregosa *et al*. 2011).

#### Identification of differential dynamic couplings and post-hoc refinement

The longitudinal multi-omics dataset comprised 10 712 molecular features, including 10 341 transcripts, 303 proteins, and 68 cytokines, measured across the shared cohort of 61 subjects. Owing to the high dimensionality of the data, we first applied the 2D variance-based filtering strategy described in the Methods section to identify informative molecular features for downstream analysis.

Pairwise DTW distances were subsequently computed for all feature pairs within and across omics layers, followed by Wilcoxon rank-sum tests to identify significant DDCs. Significant DDCs were identified using the statistical procedure described in the Methods section (adjusted FDR < 0.05).

**Figure 5:**
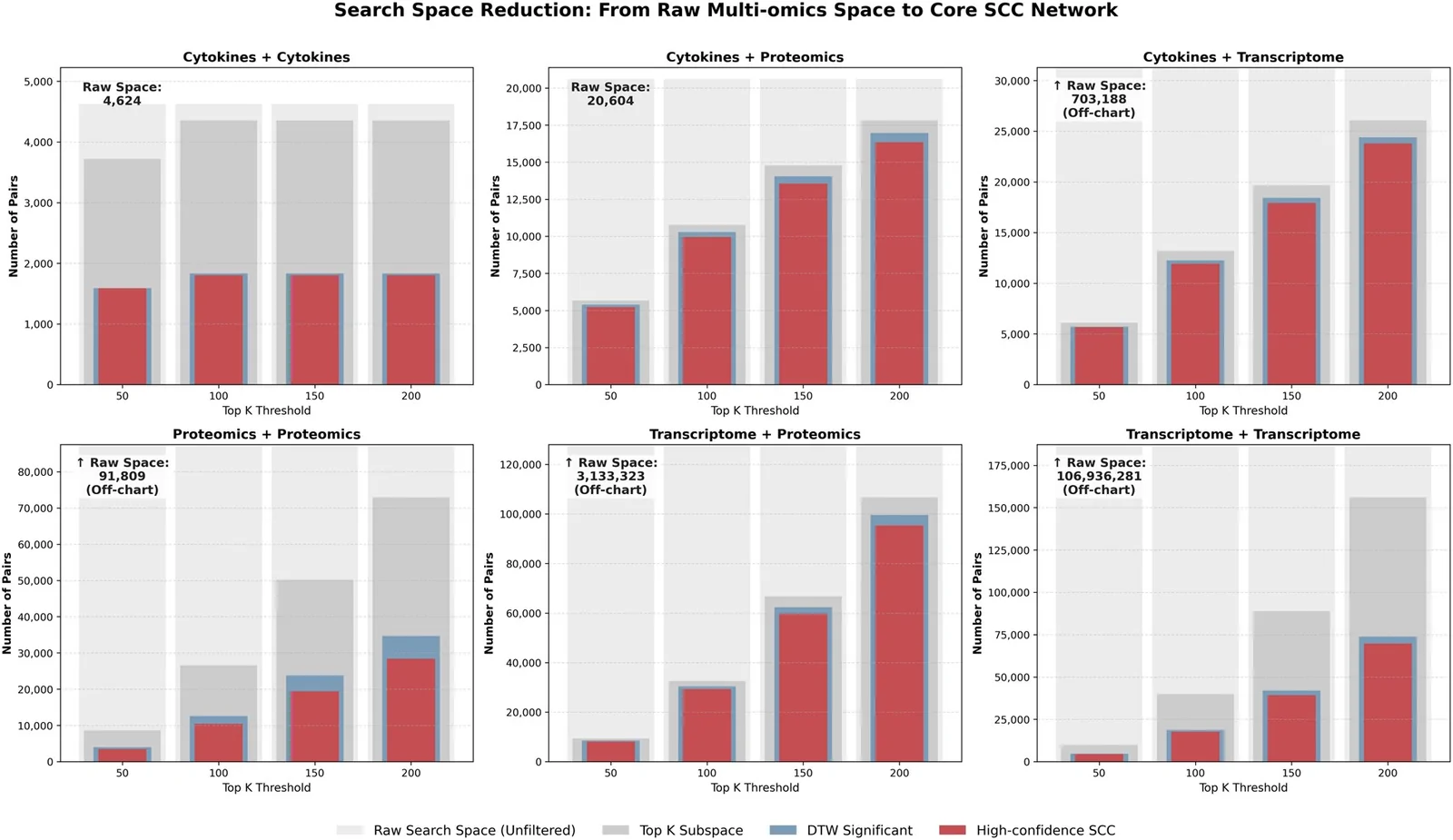
Stepwise reduction of the multi-omics search space under different feature selection thresholds. Bars represent the total unfiltered search space (light gray), the feature pairs retained after Top-*K* filtering (dark gray), statistically significant differential dynamic couplings identified by DTW (blue), and the final high-confidence Subtle Coordinated Coupling (SCC) pairs after post-hoc refinement (red). Results are shown for different feature selection thresholds (*K*).

To determine an appropriate feature selection threshold (*K*), we performed a sensitivity analysis over K∈{50,100,150,200}. The numbers of retained features obtained by taking the union of the top-*K* features ranked by temporal variance and subject-level variance within each omics layer are summarized in Fig. S3. Although the proportion of SCC pairs remained relatively stable across all threshold values, increasing *K* beyond 100 resulted in an exponential increase in the number of significant edges, substantially increasing both network complexity and computational burden (Fig. 5). Therefore, to balance signal discovery, network interpretability, computational efficiency, and the signal-to-noise ratio, we selected K=100, retaining 66 cytokines, 163 proteins, and 200 transcripts for downstream analysis (Fig. S3).

Using this analysis pipeline, we identified a total of 86 083 significant DDC pairs. Following post-hoc refinement using the linear mixed model framework, 80 970 pairs were classified as high-confidence SCCs (Fig. 5). The distribution of SCCs varied substantially across omics layers, with transcriptome–proteome interactions representing the largest group (29 256 pairs), suggesting extensive temporal coordination between gene expression and protein abundance. The post-hoc refinement further identified 5113 DTW-significant pairs (approximately 5.9% of all significant DDCs) as either Unilateral Driver DDCs or Both-Driven DDCs. These interactions are likely attributable to strong marginal changes in one or both individual molecular features rather than genuine rewiring of coordinated temporal dynamics. By removing such interactions, the proposed framework preferentially retained subtle systems-level coordination that would otherwise be masked by dominant individual feature effects.

The resulting SCC pairs were used as the edges of the final differential dynamic network, which was subsequently analysed to characterize its topological properties (Barabási and Oltvai 2004).

#### Network robustness and global topological characterization

To evaluate the robustness and reproducibility of the inferred multi-omics interactions, we performed a stability selection analysis using three stratified subsampling proportions (70%, 80%, and 90%). For each sampling proportion, 100 independent resampling iterations were conducted. In every iteration, the complete analytical pipeline, including 2D variance-based feature filtering, DTW analysis, statistical testing, and linear mixed model-based post-hoc refinement, was repeated from the beginning. To quantify interaction reproducibility, we defined an *Edge Reliability Score* (ERS) as the proportion of resampling iterations in which a given edge was consistently identified. Consequently, edges with higher ERS values represent more reproducible dynamic interactions and are less likely to arise from stochastic sampling variation.

The results demonstrate that DiDyNet exhibits high robustness across all sampling proportions (Fig. S4). Notably, the ERS consistently displayed a characteristic J-shaped distribution regardless of the subsampling proportion (70%, 80%, or 90%), with the majority of SCC edges being repeatedly identified in more than 90% of resampling iterations (Fig. S4A). This result is particularly notable because the initial 2D variance-based feature filtering is inherently sensitive to sample composition. Nevertheless, the high reproducibility of the final SCC network indicates that the identified interactions are driven by consistent cohort-level biological dynamics rather than by sample-specific artifacts.

To characterize the global topology of the resulting network, nodes were ranked according to their average degree centrality across all 100 resampling iterations. Interestingly, the highest-ranked and most stable hub nodes were exclusively derived from the proteomic layer. These proteomic hubs consistently maintained an average degree of approximately 400 across all resampling iterations (Fig. S4B), indicating that they occupy central positions within the inferred differential dynamic network and may represent key components of the underlying systems-level organization.

#### Stratified topological architecture of the core regulatory network

**Figure 6:**
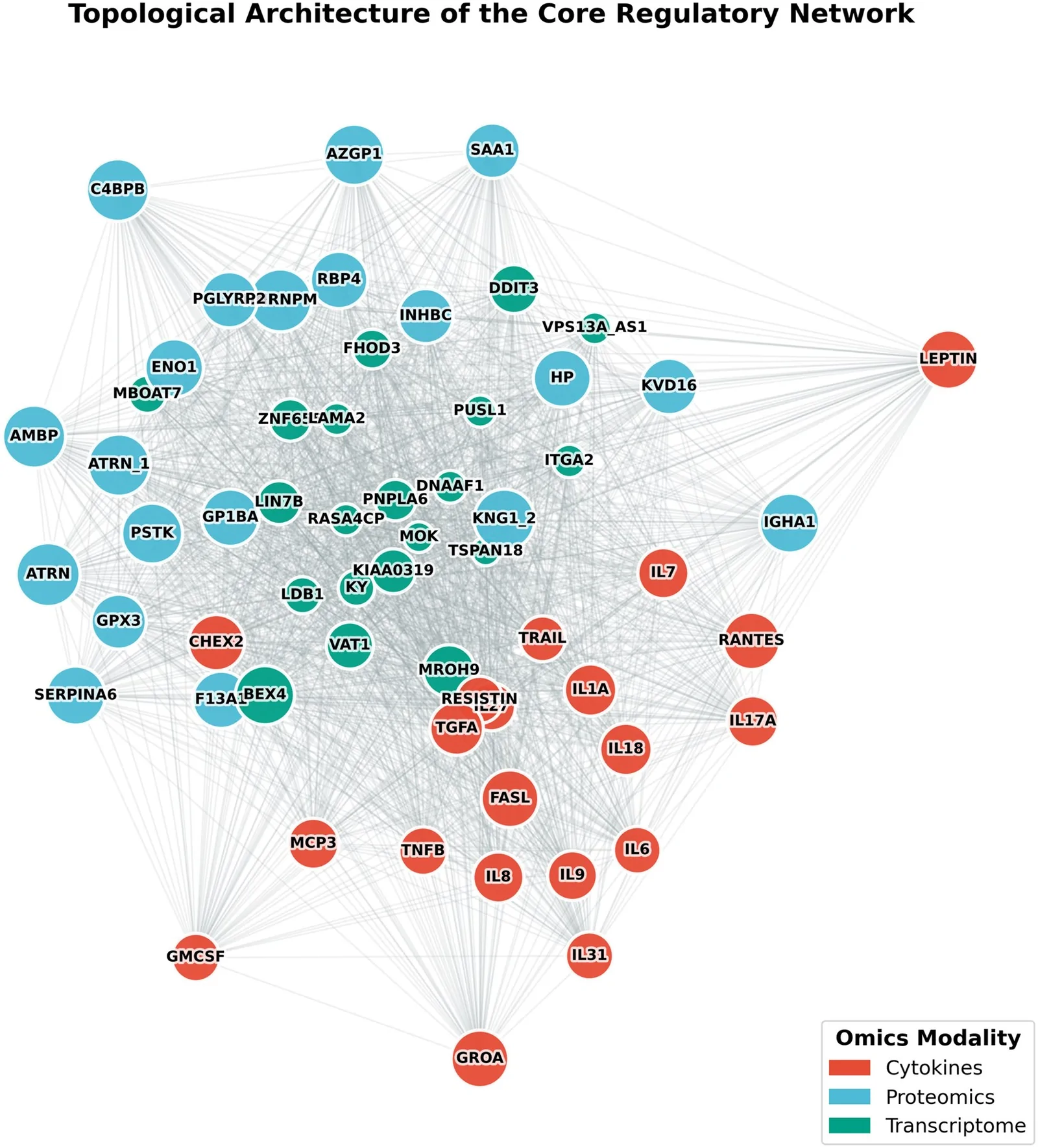
Topological architecture of the core differential dynamic network. The network displays the top 20 hub nodes from each omics layer extracted from the highly reproducible consensus network (ERS ≥ 0.9). Nodes are colored according to omics layer: cytokines (red), proteomics (blue), and transcriptomics (green). Node size is proportional to degree centrality, indicating the relative topological importance of each feature within the integrated multi-omics network. Edges represent significant high-confidence SCC relationships retained after post-hoc refinement.

Although the global network analysis effectively identifies highly connected molecular features, visualization and biological interpretation of a network of this scale are inherently limited by the “hairball effect.” Given the high topological consistency observed across all subsampling depths, we selected the network derived from the 90% subsampling fraction as the representative model for downstream biological interpretation. To improve visualization while retaining highly reproducible interactions, we constructed a consensus core network by retaining only SCC edges with an ERS of at least 0.9. To further account for the imbalance in feature abundance across different omics layers, we performed a stratified topological analysis by extracting the highest-ranked hub nodes (up to 20 nodes per omics layer) from the robust consensus network (Fig. 6). These hubs also exhibited consistently high stability across resampling iterations (Fig. S4B). Although the selected threshold was adopted primarily for visualization purposes, it can be flexibly adjusted within the DiDyNet framework according to specific analytical objectives. This consensus core network served as the basis for all downstream biological interpretation. The complete nomenclature of all molecular features is provided in Table S3.

Within the cytokine layer, the inferred network was dominated by well-established mediators of IR and metabolic inflammation. LEPTIN emerged as the highest-degree hub, consistent with extensive evidence identifying hyperleptinemia and leptin resistance as central mechanisms underlying IR and metabolic syndrome (Kahn and Flier 2000). Other highly connected cytokines included GROA and RANTES, both of which are key pro-inflammatory chemokines involved in immune-cell recruitment. In particular, the RANTES/CCR5 signaling axis has been shown to promote adipose tissue inflammation and systemic IR (Kitade *et al*. 2012), whereas CXCL1 contributes to diet-induced IR and pancreatic islet dysfunction (Nunemaker *et al*. 2014, Yoon *et al*. 2021). FASL also ranked among the major hubs, consistent with previous studies demonstrating that FasL-mediated apoptotic signaling contributes to obesity-associated IR through myeloid-cell regulation (Wueest *et al*. 2014). Interestingly, TGFA was also identified as a highly connected and reproducible hub. Although TGFA has been implicated in tissue remodeling and fibrogenesis, its systemic role in human IR remains relatively unexplored.

Within the proteomic layer, the network architecture was characterized by a prominent acute-phase and complement-related signature associated with metabolic dysfunction. C4BPB emerged as one of the most highly connected hubs, consistent with the established role of complement dysregulation in adipose inflammation and progressive IR (Phieler *et al*. 2013). Additional highly connected proteins included ATRN (together with its variant ATRN_1), AMBP, and HNRNPM, all of which have previously been associated with obesity, oxidative stress, or non-alcoholic fatty liver disease. For example, HNRNPM has been reported as a downstream target of advanced glycation end-products in lipotoxic environments (Takino *et al*. 2015). However, their central topological roles within longitudinal insulin-resistant molecular networks have received relatively little attention. The independent identification of multiple ATRN isoforms further supports the robustness of the inferred network while highlighting these proteins as promising blood-based candidates for future investigation of IR progression.

The transcriptomic layer primarily captured intracellular stress responses associated with metabolic dysfunction. *DDIT3*, a master regulator of endoplasmic reticulum stress, emerged as one of the most highly connected hubs. Previous studies have demonstrated that *DDIT3* contributes to pancreatic β-cell apoptosis, lipotoxicity, and hepatic IR during the progression of T2D (Yong *et al*. 2021). Its prominent network position therefore links the inferred transcriptomic network to well-characterized intracellular stress pathways involved in IR. In contrast, several of the remaining highly connected hubs, including *BEX4*, *MROH9*, *VAT1*, and *KIAA0319*, have received little attention in the context of metabolic syndrome or IR. Their emergence as central nodes within the differential dynamic network highlights the ability of DiDyNet to prioritize potentially important molecular candidates that warrant further functional investigation.

## Discussion

In this study, we introduced DiDyNet, a computational framework designed to characterize time-evolving molecular interactions underlying complex disease progression. Unlike conventional approaches that rely on static snapshots or differential analysis of individual molecular features, DiDyNet explicitly models temporal coordination between longitudinal molecular trajectories. By integrating DTW with a rigorous post-hoc statistical refinement strategy, the framework effectively accommodates asynchronous biological signals while reducing the influence of high-dimensional noise. Applications to both simulated datasets and a longitudinal multi-omics cohort of IR demonstrated that DiDyNet is capable of identifying subtle network rewiring events that are difficult to detect using conventional linear or static approaches.

The selection of DTW as the computational core of DiDyNet, rather than deep learning-based approaches, was motivated by the characteristics of longitudinal clinical multi-omics data. Although deep learning methods have achieved considerable success in general time-series analysis, their application to clinical multi-omics studies is often constrained by the combination of high dimensionality, limited sample size, and relatively short longitudinal follow-up. Under these conditions, highly parameterized models are susceptible to overfitting and frequently require substantially larger training datasets. In contrast, DTW provides a robust, non-parametric measure of trajectory similarity that requires minimal parameter tuning while naturally accommodating temporal misalignment. Moreover, interpretability is particularly important in biomarker discovery and systems biology. Unlike black-box deep learning models, DTW explicitly identifies the optimal alignment between temporal trajectories, providing intuitive insights into temporal delays and coordinated molecular dynamics.

A central methodological contribution of DiDyNet is the integration of DTW-based trajectory alignment with LMM-based post-hoc refinement. While DTW effectively captures nonlinear and asynchronous temporal relationships, it does not distinguish genuine coordinated dynamics from apparent associations driven by large marginal changes in individual features. By incorporating LMM as an independent post-hoc refinement step, DiDyNet separates coordinated temporal rewiring from dominant single-feature effects, thereby improving the biological interpretability of the inferred network.

From a biological perspective, the differential dynamic network identified by DiDyNet revealed a coordinated systems-level landscape associated with IR. The prominent network positions of LEPTIN together with the inflammatory chemokines GROA and RANTES are consistent with previous studies highlighting chronic metabolic inflammation and immune-cell recruitment as major components of IR. The identification of FASL and TGFA further suggests coordinated interactions involving apoptotic signaling and tissue remodeling during disease progression. Within the proteomic layer, highly connected hubs including C4BPB and ATRN indicate extensive complement activation and acute-phase responses associated with metabolic dysfunction. At the transcriptomic level, *DDIT3* emerged as one of the principal network hubs, linking the inferred network to established endoplasmic reticulum stress pathways involved in IR. In addition, several highly connected genes, including *BEX4*, *MROH9*, and *VAT1*, remain relatively unexplored in the context of metabolic syndrome. Their prominent positions within the inferred differential dynamic network suggest that they represent promising candidates for future functional investigation.

Despite these encouraging results, several limitations should be acknowledged. First, although DTW effectively accommodates temporal misalignment, pairwise trajectory alignment remains computationally intensive because of its quadratic time complexity ($O(N^{2})$), potentially limiting scalability to extremely large datasets such as single-cell longitudinal studies without further optimization. Second, the DCs identified by DiDyNet represent statistical associations rather than causal relationships. Establishing causal regulatory mechanisms will require experimental perturbation studies or integration with causal inference frameworks. Third, the current post-hoc refinement relies on LMMs, which provide an effective baseline filter for linear temporal trends but may not fully account for more complex nonlinear baseline behaviors. Future extensions could incorporate nonlinear mixed-effects models or generalized additive mixed models to provide greater flexibility in modeling individual molecular trajectories. Finally, the present framework classifies feature dynamics into discrete categories during post-hoc refinement. Future developments may incorporate Bayesian probabilistic approaches to quantify uncertainty associated with these classifications.

Overall, DiDyNet provides a statistically rigorous and interpretable framework for differential dynamic network inference from longitudinal multi-omics data. By explicitly modeling coordinated temporal dynamics across molecular layers, it offers a complementary perspective to conventional differential expression and static network analyses and provides a general framework for investigating dynamic molecular regulation in complex diseases.

## Supplementary Material

vbag192_Supplementary_Data

## Data Availability

The longitudinal multi-omics dataset analysed in this study is publicly available from the study of Sailani *et al*. (2020).
