# Supplementary material for: DiDyNet: a robust framework for differential dynamic network inference from longitudinal multi-omics data
Source: Bioinform Adv. 2026 Jul 30;6(1):vbag192. doi: 10.1093/bioadv/vbag192 (PMC13493046; doi:10.1093/bioadv/vbag192)
Supplement: Supplementary file 1 [file vbag192_supplementary_data.docx]

**Supplementary Material**

**Table S1 Candidate Coupling Functions used in the DDC generation model.**

| **Name** | **Mathematical Expression** | **Description** |
| --- | --- | --- |
| Sine | f (x) = sin(x) | Periodic oscillation |
| Exponential | f (x) = $e^{x}$ | Rapid growth |
| Sigmoid | f (x) =$\frac{1}{1+e^{-x}}$ | S-shaped saturation |
| Cubic | f (x) = $x^{3}$ | Polynomial growth |
| Tanh | f (x) = tanh(x) | Symmetric saturation |
| Mixed Sine | f (x) = 0.5 x + 0.5 sin(x) | Linear with fluctuation |
| Mixed Exp | f (x) = 0.5 x + 0.5 $e^{x}$ | Linear-Exponential mix |
| Mixed Sigmoid | f (x) = 0.5 x + $\frac{0.5}{1+e^{-x}}$ | Linear-Sigmoid mix |
| Mixed Square | f (x) = 0.5 x + 0.5 $x^{2}$ | Linear-Quadratic mix |
| Mixed Cubic | f (x) = 0.5 x + 0.5 $x^{3}$ | Linear-Cubic mix |

**Table S2** Parameter configurations for sensitivity analysis.

| **Parameter** | **Tested Values** |
| --- | --- |
| Number of features (P) | 20, 40, 60, 80, 100 |
| Number of true DDCs | 1, 3, 5, 8, 10 |
| Coupling Strength Contrast (ρA vs. ρB ) | (0.9, 0.1), (0.8, 0.2), (0.7, 0.3), (0.6, 0.4), (0.5, 0.5) |
| Sample Size per group (N) | 10, 20, 30, 40, 50 |
| Time Points (T) | 3, 5, 8, 10, 12 |
| Noise Level | 0.1, 0.2, 0.3, 0.4, 0.5 |

**Table S3.** List of abbreviations and full names for all molecular features mentioned in the manuscript, sorted alphabetically

| **Abbreviation** | **Full Name** |
| --- | --- |
| **AMBP** | Alpha-1-Microglobulin/Bikunin Precursor |
| **ATRN** | Attractin |
| **ATRN_1** | Attractin (variant 1) |
| **BEX4** | Brain Expressed X-Linked 4 |
| **C4BPB** | Complement C4b-Binding Protein Beta chain |
| **CCR5** | C-C Motif Chemokine Receptor 5 |
| **DDIT3** | DNA Damage Inducible Transcript 3 |
| **FASL** | Fas Ligand |
| **GROA** | Growth-Regulated Alpha Protein (CXCL1) |
| **HNRNPM** | Heterogeneous Nuclear Ribonucleoprotein M |
| **KIAA0319** | KIAA0319 |
| **LEPTIN** | Leptin |
| **MROH9** | Maestro Heat-Like Repeat Family Member 9 |
| **RANTES** | Regulated upon Activation, Normal T Cell Expressed and Presumably Secreted (CCL5) |
| **TGFA** | Transforming Growth Factor Alpha |
| **VAT1** | Vesicle Amine Transport 1 |

**Figure S1. Comprehensive multi-omics data preprocessing and feature selection workflow.**

**Figure S2 Availability of multi-omics data across subjects and time points.** Each point represents a subject at a specific time point, color-coded by the number of available omics layers: blue for three, orange for two, and red for one.

**Figure S3. Variance-based feature screening strategy.** Features were filtered based on their dynamic range across time (X-axis) and subjects (Y-axis). Panels show the selection results for Cytokines, Transcriptomics, and Proteomics under different Top- thresholds. Blue dots denote the selected features (Union of Top- Temporal and Top- Subject variance).

**Figure S4 Stability and topological robustness of the multi-omics SCC network across varying subsampling depths. (A) Distribution of edge selection frequencies.** The consistent J-shaped distributions across 100 resampling runs at multiple stratified subsampling fractions (70%, 80%, and 90%) indicate that the majority of identified SCCs are highly stable and invariant to patient-level perturbations. **(B) Distribution of average node degrees.** Across all subsampling thresholds, the network exhibits a robust hub-based architecture, where a core subset of "super hubs" consistently maintains high connectivity (average degree > 300) regardless of the sample exclusion rate.
